# Comparison of Experimental Rat Models in Donation After Circulatory Death (DCD): *in-situ* vs. *ex-situ* Ischemia

**DOI:** 10.3389/fcvm.2020.596883

**Published:** 2021-01-13

**Authors:** Maria Arnold, Natalia Méndez-Carmona, Rahel K. Wyss, Anna Joachimbauer, Daniela Casoni, Thierry Carrel, Sarah Longnus

**Affiliations:** ^1^Department of Cardiovascular Surgery, Inselspital, Bern University Hospital and Department for BioMedical Research (DBMR), University of Bern, Bern, Switzerland; ^2^Experimental Surgery Facility (ESF), Department for BioMedical Research (DBMR), University of Bern, Bern, Switzerland

**Keywords:** cardiac ischemia-reperfusion, experimental rat models, donation after circulatory death (DCD), *ex-situ* ischemia, *in-situ* ischemia, heart transplantation

## Abstract

**Introduction:** Donation after circulatory death (DCD) could substantially improve donor heart availability. However, warm ischemia prior to procurement is of particular concern for cardiac graft quality. We describe a rat model of DCD with *in-situ* ischemia in order to characterize the physiologic changes during the withdrawal period before graft procurement, to determine effects of cardioplegic graft storage, and to evaluate the post-ischemic cardiac recovery in comparison with an established *ex-situ* ischemia model.

**Methods:** Following general anesthesia in male, Wistar rats (404 ± 24 g, *n* = 25), withdrawal of life-sustaining therapy was simulated by diaphragm transection. Hearts underwent no ischemia or 27 min *in-situ* ischemia and were explanted. *Ex situ*, hearts were subjected to a cardioplegic flush and 15 min cold storage or not, and 60 min reperfusion. Cardiac recovery was determined and compared to published results of an entirely *ex-situ* ischemia model (*n* = 18).

**Results:** In donors, hearts were subjected to hypoxia and hemodynamic changes, as well as increased levels of circulating catecholamines and free fatty acids prior to circulatory arrest. Post-ischemic contractile recovery was significantly lower in the *in-situ* ischemia model compared to the *ex-situ* model, and the addition of cardioplegic storage improved developed pressure-heart rate product, but not cardiac output.

**Conclusion:** The *in-situ* model provides insight into conditions to which the heart is exposed before procurement. Compared to an entirely *ex-situ* ischemia model, hearts of the *in-situ* model demonstrated a lower post-ischemic functional recovery, potentially due to systemic changes prior to ischemia, which are partially abrogated by cardioplegic graft storage.

## Introduction

Grafts obtained from donation after circulatory death (DCD) could substantially improve donor heart availability, which is a major issue in heart transplantation. However, concerns related to graft quality persist as they are subjected to a period of warm global ischemia before procurement. Despite excellent clinical outcomes in DCD heart transplantation (1, 2), further expansion of the donor pool requires the optimization of protocols to guarantee optimal graft quality.

In order to evaluate strategies for improving current DCD protocols, the choice of the most appropriate and clinically relevant animal models is essential. The *ex-situ* working rat heart system is a well-established experimental model that has been used in several studies to investigate cardioprotective strategies and ischemic tolerance in the context of DCD (3–8). With this model, the pre-ischemic and ischemic phases are carried out in the *ex-situ* heart preparation. This model allows tight control of experimental conditions, such as the precise timing of warm ischemic onset, and pre-ischemic energy substrate availability. Further, the *ex-situ* preparation permits detailed functional and biochemical assessment prior to warm ischemia and reperfusion. However, this model lacks the physiological changes that occur after the withdrawal of life sustaining therapy (WLST) in the donor. (9, 10). This is of particular importance in DCD, as the heart is not only exposed to warm ischemia, but also to a potentially damaging pre-ischemic phase prior to procurement.

The consideration of this pre-ischemic phase and the simulation of the DCD process in a donor is receiving more and more attention in the field, and several *in-situ* rat models have recently been developed (11–20). However, no standard protocol is available; for example, initiation of the DCD process can be simulated in different ways; by simply terminating the ventilatory support (14, 15, 17), by terminating ventilatory support in combination with a tracheal clamp (12, 13), with or without the addition of respiratory muscle paralysis (11, 16), or by the transection of the diaphragm (19, 20). Furthermore, not all protocols employ the same definition of the start of warm ischemia. For example, some measure cardiac asystole by electrocardiogram (11) while others define the beginning of ischemia when the peak systolic pressure drops below 30 mmHg or asystole (12, 13), or when the blood pressure was non-pulsatile or the mean arterial pressure less than 30 mmHg (14, 17). As such, strict comparison among varying pre-clinical models requires careful interpretation, but when possible, it may aid in advancing our understanding of the pathophysiologic processes involved.

Post-ischemic cardiac function, as well as the efficacy of protective strategies may not only be influenced by ischemia itself, but also by pre-ischemic conditions (9), which can alter cardiac metabolism upon reperfusion. Studies in pre-clinical models have shown that the phase between WLST and ischemia is characterized by hemodynamic instability, pressure and volume overload, significant drop up to cessation of arterial pulsatility and subsequent systemic responses that lead to catecholamine release (9, 10, 17). Besides influencing cardiac rhythm and contractility, catecholamines are also known to trigger the release of free fatty acids (FFA) into the circulation (21). Although increased pre-ischemic concentrations of FFA have not yet been measured in DCD, our group has demonstrated that pre-ischemic substrate availability, e.g., acute expose to high circulating fatty acids, can negatively affect post-ischemic cardiac recovery (22).

As interventions prior to circulatory death are limited for ethical reasons, strategies to reduce post-ischemic cardiac dysfunction that are applied after heart procurement hold much promise, particularly the initial reperfusion after warm ischemia. The majority of centers performing DCD heart transplantation are currently using St. Thomas' N°2 cardioplegia or Bretschneider histidine–tryptophan–ketoglutarate (HTK) crystalloid solution (Custodiol) supplemented with erythropoietin and glyceryl-trinitrate (23). However, the impact of perfusion with cold preservation solutions and this brief period of cold ischemia on post-ischemic functional recovery has not yet been investigated.

With the current study, we aimed to establish a clinically relevant *in-situ* ischemia rat model of DCD in our lab, to characterize the period between WLST until the end of functional, warm ischemia, and to evaluate the effects of cold cardioplegic storage in the *in-situ* model. Furthermore, we evaluated and compared post-ischemic cardiac recovery with isolated hearts from a published *ex-situ* ischemia model (6), using new measurements and analyses.

## Materials and Methods

### Ethics Statement

All experimental procedures were performed in compliance with the European Convention for Animal Care and approved by the Swiss animal welfare authorities and the Ethics Committee for Animal Experimentation, Berne, Switzerland (Veterinärdienst des Kantons Bern; BE40/15, BE103/17 and BE104/18). Surgery was performed under anesthesia, and all efforts were made to minimize animal suffering.

### Animals

Male Wistar rats (Janvier Labs, Le Genest-Saint-Isle, France) were housed in groups under standard conditions with a 12 h light-dark cycle at a controlled room temperature, humidity and *ad libitum* access to water and food (KLIBA NAFAG, GRANOVIT AG, Kaiseraugst, Switzerland). Rats weighing 375–425 g and aged 11–12 weeks were chosen to represent young-adult, human DCD heart donors and to ensure mature cardiac metabolism (24, 25). A total of 43 rats were used in this study, and 7 rats over 6 experimental groups were excluded due to technical issues in cardiac function recording or long episodes of fibrillation (Supplementary Figure 3). The total number of rats included in each group are reported in Tables 1, 2.

**Table 1:** Baseline characteristics of *ex-situ* hearts.

	**NI-ES**	**I-ES**
Number of hearts	8	7
Body weight (g)	406 ± 31	390 ± 19
Heart rate (beats * min^−1^)	258 ± 41	255 ± 39
Left ventricular work (mmHg*beats*min^−1*^10^3^)	33 ± 4	34 ± 5
Developed pressure (mmHg)	129 ± 13	132 ± 10
dP/dt_min_ (mmHg*s^−1*^10^3^)	−4.5 ± 1.0	−4.3 ± 0.6
dP/dt_max_ (mmHg*s^−1*^10^3^)	4.4 ± 0.6	4.3 ± 0.4
Cardiac output (mL*min^−1^)	63 ± 11	67 ± 11
Coronary flow (mL*min^−1^)	32 ± 5	32 ± 4

*Cardiac functional parameters were obtained during the 20-min perfusion (average of 10 and 20 min values) before ischemia in isolated, working hearts. dP/dt max, maximum first derivative of LV pressure; dP/dt min, minimum first derivative of LV pressure; I-ES, ischemic ex-situ model; NI-ES, non-ischemic ex-situ model*.

**Table 2:** Baseline characteristics of *in-situ* hearts.

	**NI-IS**	**NI-IS+**	**I-IS**	**I-IS+**
Number of hearts	5	5	6	5
Body weight (g)	397 ± 24	394 ± 15	418 ± 26	405 ± 28
Heart rate (beats * min^−1^)	224 ± 9	234 ± 43	249 ± 37	255 ± 12
Peak systolic pressure (mmHg)	95 ± 15	93 ± 10	107 ± 11	104 ± 5
Pulse pressure (mmHg)	29 ± 16	17 ± 11	31 ± 13	29 ± 5

*Heart rate, peak systolic pressure and pulse pressure were measured before withdrawal of life sustaining therapy in vivo. I-IS, ischemic in-situ model; I-IS+, ischemic in-situ model with cardioplegia; NI-IS, non-ischemic in-situ model; NI-IS+, non-ischemic in-situ model with cardioplegia*.

### Experimental Set Up

**Figure 1:**
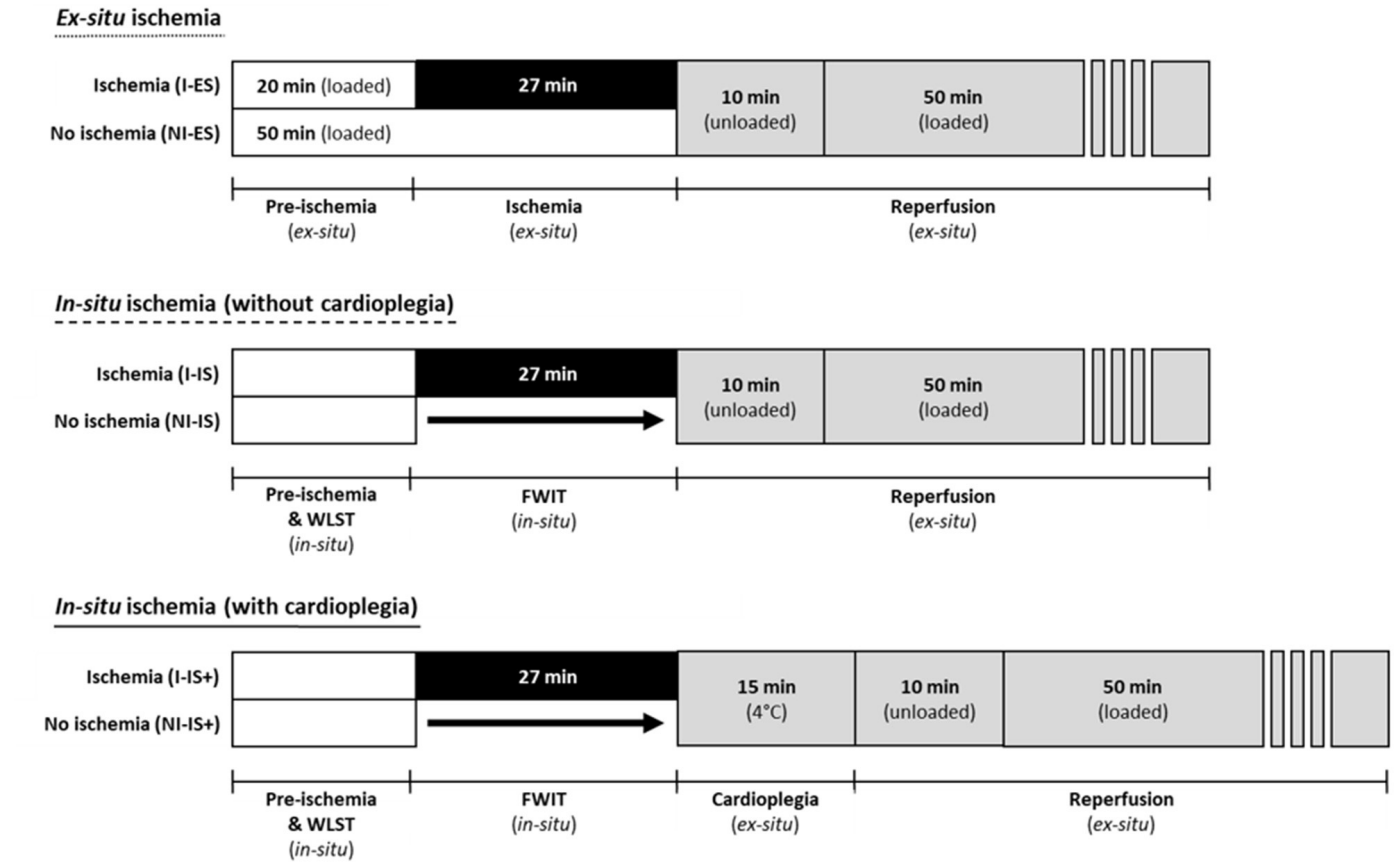
Perfusion protocols for all experimental groups using the *ex-situ* model and the *in-situ* model with and without cardioplegic storage. *Ex-situ* ischemia was induced by clamping pre- and afterload lines. *In-situ* ischemia/functional warm ischemia (FWIT) was induced by simulating withdrawal of life sustaining therapy (WLST). FWIT start was defined as peak systolic pressure <50 mmHg and FWIT end was defined as initial coronary perfusion (with cardioplegia or physiologic buffer).

We established and characterized an *in-situ* rat model of DCD in our lab. With this model, four different experimental conditions were investigated: 0 and 27 min *in-situ* ischemia (IS), and 0 and 27 min *in-situ* ischemia with the addition of cardioplegia and cold storage (IS+). Post-ischemic cardiac recovery was determined following heart explantation and perfusion under normothermic conditions for 60 min. Cardiac recovery was also compared to recently published corresponding ischemic groups generated with a purely *ex-situ* ischemia model (ES) (6) following further measurements and analyses. The protocols of all six experimental groups are presented in Figure 1. Hearts of all six experimental groups were then reperfused *ex situ* to determine post-ischemic cardiac recovery.

#### Heart Preparation for Ischemia *in-situ*

Surgical anesthesia was induced in rats with an intraperitoneal injection of 78 mg/kg ketamine (Narketan®, Vetoquinol AG, Bern, Switzerland), 7.2 mg/kg xylazine (Xylapan®, Vetoquinol AG, Bern, Switzerland), and 1.2 mg/kg acepromazine (Prequillan, Fatro AG, Bologna, Italy). Arterial oxygen saturation (SaO_2_) was monitored using a toe-clip pulse oximeter (AD Instruments, Spechbach, Germany). Animals received 100% oxygen via a face mask and flow adjustments were made to keep SaO_2_ between 95 and 99%. When the hind-paw withdrawal reflex could not be elicited after toe pinch, a catheter was inserted into the right common carotid artery and connected to a pressure transducer for continuous recording of blood pressure and heart rate. Seven hundred and fifty U.I./kg of sodium heparin (Liquemin®, Drossapharm AG, Arlesheim, Switzerland) were injected through this catheter, which also served as blood sampling port. Withdrawal of life sustaining therapy was simulated by asphyxiation through transection of the diaphragm. Functional warm ischemic time (FWIT) was considered to start when the peak systolic pressure dropped below 50 mmHg. Circulatory arrest was declared when the pulse pressure was lower than 3 mmHg. Body temperature was measured by introducing a sensor tip thermometer into the thoracic cavity and temperature was kept between 36.6 and 37.2°C using a heating lamp (if required). After 27 min of ischemia, all hearts were explanted, and aortae cannulated in the same isolated heart system used with the *ex-situ* ischemia model for the reperfusion period where cardioplegia was administered following the current clinical practice: hearts were flushed with 7 mL ice cold St. Thomas' N°2 cardioplegic solution supplemented with 100 mg/mL glyceryl trinitrate (GTN; Nitroglycerin Bioren, Sintetica, Mendrisio, Switzerland) and 5,000 U/L erythropoietin (EPO; Eprex, Janssen, Berchem, Belgium) (26) at a constant pressure of 60 mmHg and then statically stored for a total of 15 min immersed in ice-cold St. Thomas' N°2 cardioplegia. FWIT was considered to end with the initiation of the cardioplegic flush.

#### Heart Preparation for Ischemia *ex-situ*

Surgical anesthesia was induced in rats with an intraperitoneal injection of 100 mg/kg ketamine (Narketan®, Vetoquinol AG, Bern, Switzerland), and 10 mg/kg xylazine (Xylapan®, Vetoquinol AG, Bern, Switzerland). Adequate depth of anesthesia was confirmed by absence of the hind-paw withdrawal reflex. Hearts were then rapidly excised, placed in ice-cold phosphate-buffered saline, and working- mode preparations were established. Isolated, working rat hearts were prepared as previously described (5, 6). Briefly, hearts were aerobically perfused with a modified Krebs-Henseleit buffer containing 1.25 mM Ca^2+^, 11 mM glucose, 1.2 mM palmitate bound to 3% BSA and 0.5 mM lactate oxygenated with 95% O_2_-5% CO_2_ for a pre-ischemic period of 20 min at a constant pressure of 80 mmHg. Ischemia of 27 min was initiated and terminated by clamping and unclamping of the pre- and after- load perfusion lines, respectively. During the ischemic period, the heart was immersed in a glucose- and palmitate-free Krebs-Henseleit buffer at 37°C.

#### Protocol for Post-ischemic Heart Perfusions (Both Models)

Ischemic and non-ischemic hearts from both models were reperfused for a total of 60 min using a modified Krebs-Henseleit buffer containing 1.25 mM Ca^2+^ and 11 mM glucose. Hearts remained unloaded during the first 10 min. During this time, the left atria of hearts from the *in-situ* model were cannulated. The perfusion was then switched to working mode for another 50 min. During the 60 min reperfusion, perfusate was recirculating, except the first minute of reperfusion in hearts obtained with the *in-situ* model, when cardioplegia or blood was washed out and discarded. All hearts were maintained at 37°C with an aortic pressure of 60 mmHg. At the end of the protocol, hearts were immediately frozen in liquid nitrogen and stored at −80°C.

#### Assessment of Cardiac Function

Intraventricular cardiac pressure was measured using a micro-tip pressure catheter (Millar, Houston, USA) placed in the left ventricle. Perfusate flow was measured using flow probes (Transonic Systems Inc., Ithaca, USA) placed in pre- and after- load lines. Data were continuously recorded using a PowerLab data acquisition system (ADInstruments, Spechbach, Germany).

#### Blood and Perfusate Sampling

During the *in-situ* ischemia protocol, blood analysis was performed before withdrawal, during the withdrawal phase (FWIT and cardiac arrest time points) and, for the ischemic hearts, at the end of the 27 min *in-situ* ischemia. Blood samples were obtained from the carotid artery (arterial blood) prior to heart procurement, and from the chest cavity after heart procurement at end-ischemia (mixed arterial-venous blood). Samples were used for blood gas analysis (see details in the Supplementary Material) and the remainder was transferred to lithium heparin tubes and centrifuged at 2,000g for 5 min. Plasma was stored at −80°C.

During post-ischemic heart perfusions, coronary effluent and circulating perfusate were sampled at multiple time points. Samples were stored at −80°C.

#### Additional Methods

The following methods and materials are described in detail in the Supplementary Material: measurement of blood gas, circulating factors and measurement of tissue water content.

### Statistical Analysis

Values are reported as mean ± standard deviation or as median, 25–75 percentiles and range (box-and-whiskers). Statistical analyses were performed with GraphPad Prism software (GraphPad Software, Inc., La Jolla, CA). The Kruskal-Wallis test was performed for an overview of differences between experimental groups and, when significant overall results were observed, comparisons between groups of interest were performed with Mann-Whitney analyses. Two-tailed *p*-values were adjusted for multiple comparisons (modified, sequential, rejective Bonferroni procedure) (27). Corrected *p*-values are reported and considered statistically significant if <0.05.

## Results

### Baseline Characteristics

Baseline characteristics for the ischemic *ex-situ* and *in-situ* groups are represented in Tables 1, 2, respectively. No difference in baseline characteristics among experimental groups was observed in either the *ex-situ* or the *in-situ* model.

### Withdrawal Phase (*in-situ* Ischemia Model)

The average time from WLST to FWIT start was 1.0 ± 0.6 min. For hearts that underwent ischemia, mean time from FWIT start to circulatory arrest was 1.9 ± 0.9 min. No difference among experimental groups was observed (Supplementary Table 1).

**Figure 2:**
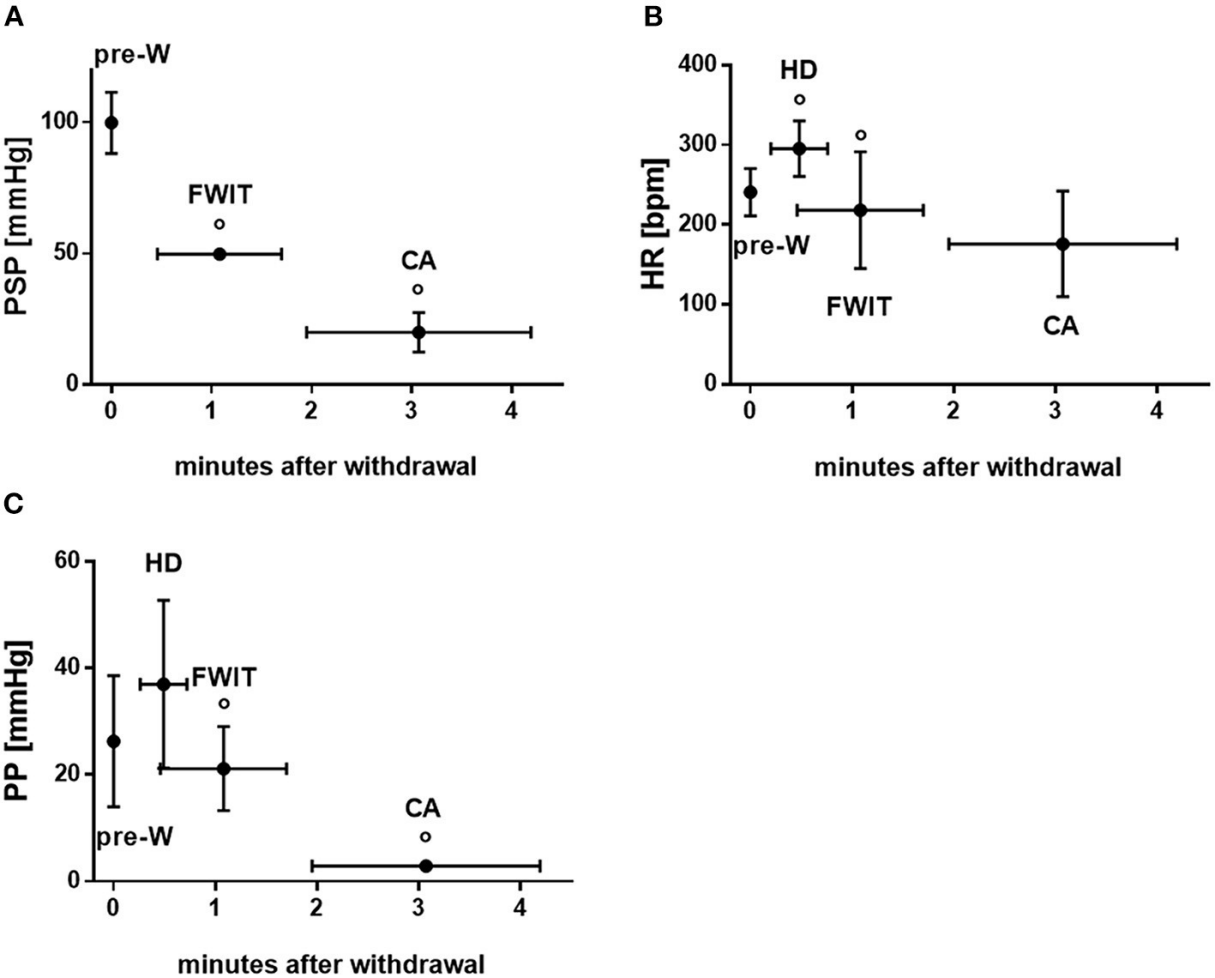
Functional parameters during *in-situ*/withdrawal phase. **(A)** Peak systolic pressure (PSP) **(B)** heart rate (HR) **(C)** pulse pressure (PP). CA, circulatory arrest; FWIT, functional warm ischemia start; HD, hyperdynamic phase; pre-W, pre-withdrawal of life sustaining therapy. Data are expressed as mean ± standard deviation. Statistical analyses were only performed between consecutive time points. °*p* < 0.05 vs. corresponding previous time point. *n* = 10–21/time point.

Peak systolic pressure in rats dropped significantly after WLST and was, per definition, 50 mmHg at FWIT start (*p* < 0.05; Figure 2A). A hyperdynamic phase was observed for heart rate and pulse pressure. Heart rate increased after WLST (*p* < 0.05) before it finally dropped (Figure 2B). Similarly, pulse pressure appeared to increase after WLST, however this change did not reach statistical significance. Subsequently, pulse pressure continuously dropped to 3 mmHg, at which time circulatory arrest was declared (*p* < 0.05 hyperdynamic phase vs. FWIT and FWIT vs. cardiac arrest; Figure 2C).

**Figure 3:**
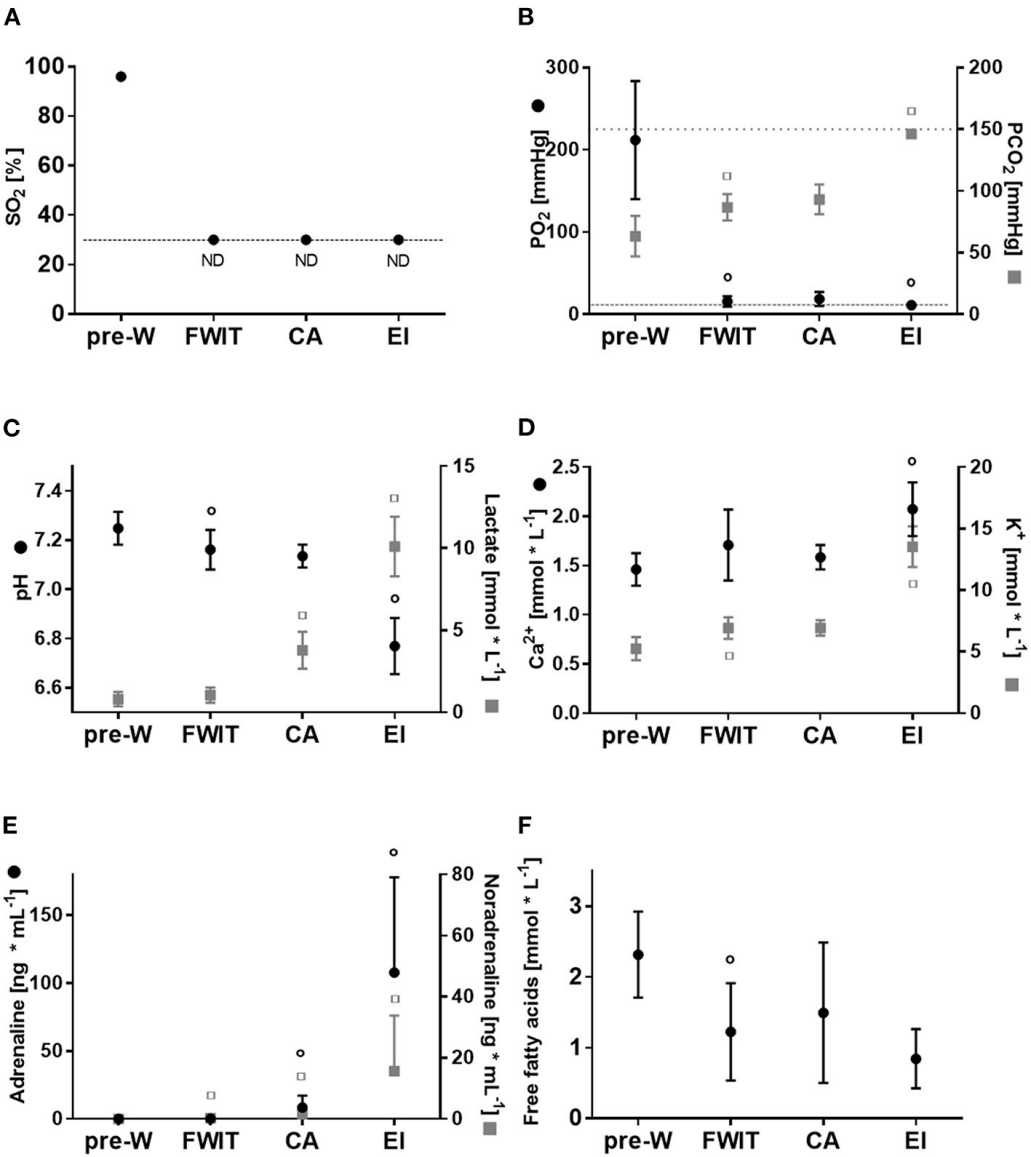
Blood parameters during *in-situ*/withdrawal phase. **(A)** Oxygen saturation (SO_2_) **(B)** partial oxygen and carbon dioxide pressure (PO_2_ and PCO_2_) **(C)** blood pH and lactate **(D)** blood calcium (Ca^2+^) and potassium (K^+^) **(E)** blood adrenaline and noradrenaline **(F)** blood free fatty acids. CA, circulatory arrest; EI, end-ischemia; FWIT, functional warm ischemia start; ND, below detection limit; pre-W, pre-withdrawal of life sustaining therapy. Data are expressed as mean ± standard deviation. Statistical analyses were only performed between consecutive time points. ° or ^□^ *p* < 0.05 vs. corresponding previous time point. *n* = 8–21/time point.

Blood O_2_ saturation immediately dropped after WLST and was below the detection level (<30%) for all subsequent measurement time points (Figure 3A). A similar steep decline was observed for the partial pressure of oxygen (PO_2_), as shown in Figure 3B. Partial pressure of carbon dioxide (PCO_2_) at the pre-withdrawal of life sustaining therapy time point already indicated hypercapnia (63.3 ± 16.4 mmHg). PCO_2_ after WLST increased and reached the upper detection threshold (>150 mmHg) at the end of ischemia (*p* < 0.05 cardiac arrest vs. end-ischemia; Figure 3B).

Blood pH dropped significantly from pre-withdrawal of life sustaining therapy to FWIT (*p* < 0.05), and remained stable from FWIT to cardiac arrest. At the end of 27 min ischemia, pH further decreased to a profound acidosis (*p* < 0.05; Figure 3C) with a concomitant increase in blood lactate concentration (Figure 3C).

Both blood calcium and potassium concentrations increased, being highest at end ischemia (Figure 3D). Blood sodium, chloride and glucose concentrations are presented in Supplementary Figure 1.

Concentrations of catecholamines in blood are represented in Figure 3E; adrenaline and noradrenaline both increased at FWIT to cardiac arrest (*p* < 0.05 for both; Figure 3E). Peak concentrations were reached at end ischemia.

Blood free fatty acid concentrations peaked before withdrawal and dropped significantly by FWIT (*p* < 0.05; Figure 3F).

### Post-ischemic Functional Recovery

**Figure 4:**
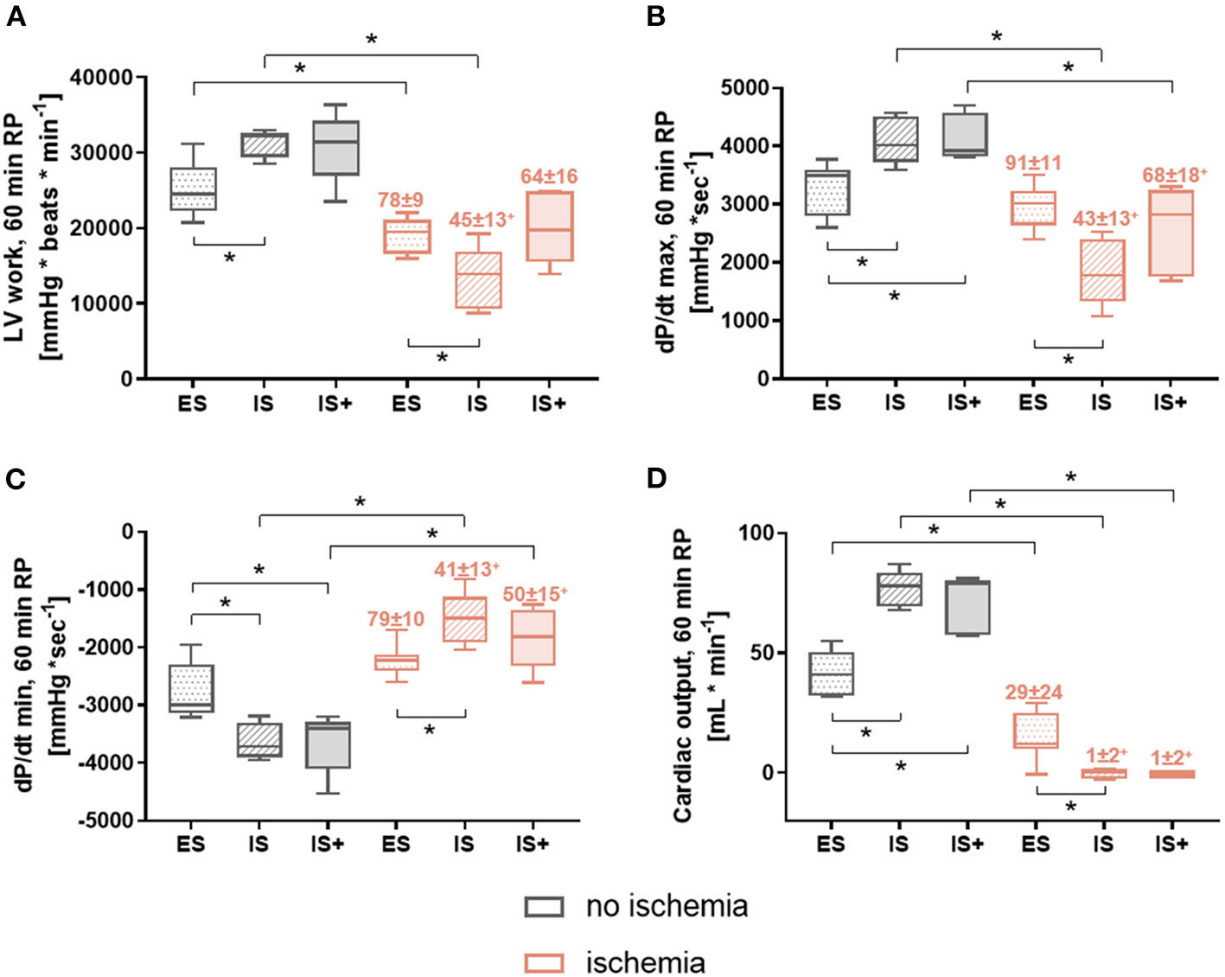
Post-ischemic cardiac function at 60 min reperfusion. **(A)** LV work (left ventricular work: heart rate*developed pressure) **(B)** dP/dt max (maximum first derivative of LV pressure) **(C)** dP/dt min (minimum first derivative of LV pressure) **(D)** CO (cardiac output). Data are expressed as median, 25–75 percentiles, and range; numbers indicate percentage of corresponding non-ischemic value. *p < 0.05, +*p* < 0.05 vs. ES. ES, *ex-situ* model; IS, *in-situ* model; IS+, *in-situ* model with cardioplegia; RP, reperfusion; *n* = 5–8 per group.

Post-ischemic functional recovery is presented in Figure 4 and Supplementary Figure 2. In general, left ventricular work at the end of reperfusion (Figure 4A) was lower after ischemia compared to the corresponding non-ischemic groups, reaching statistical significance for IS and ES groups (*p* < 0.05 for both). Furthermore, among non-ischemic hearts, left ventricular work tended to be lower in ES vs. IS and IS+ hearts, reaching statistical significance between IS and ES groups (*p* < 0.05). However, among ischemic hearts, left ventricular work was significantly decreased in IS vs. ES hearts (*p* < 0.05). Thus, recovery expressed as percentage of corresponding non-ischemic group, was significantly lower in IS vs. ES (45 ± 13% vs. 78 ± 9%, respectively, *p* < 0.05). Interestingly, no differences were observed between IS+ and ES hearts for both absolute values and percentage recovery of left ventricular work.

Similar patterns were observed for contractility rate (dP/dt max) and relaxation rate (dP/dt min) at 60 min of reperfusion (Figures 4B,C); however, although a tendency for improved recovery was observed with IS+ compared to IS hearts, the percent recovery for these hearts remained significantly lower than that of ES hearts.

The results of cardiac output (Figure 4D) were also similar; however, the addition of cardioplegia did not appear to improve recovery.

### Vascular Parameters

**Figure 5:**
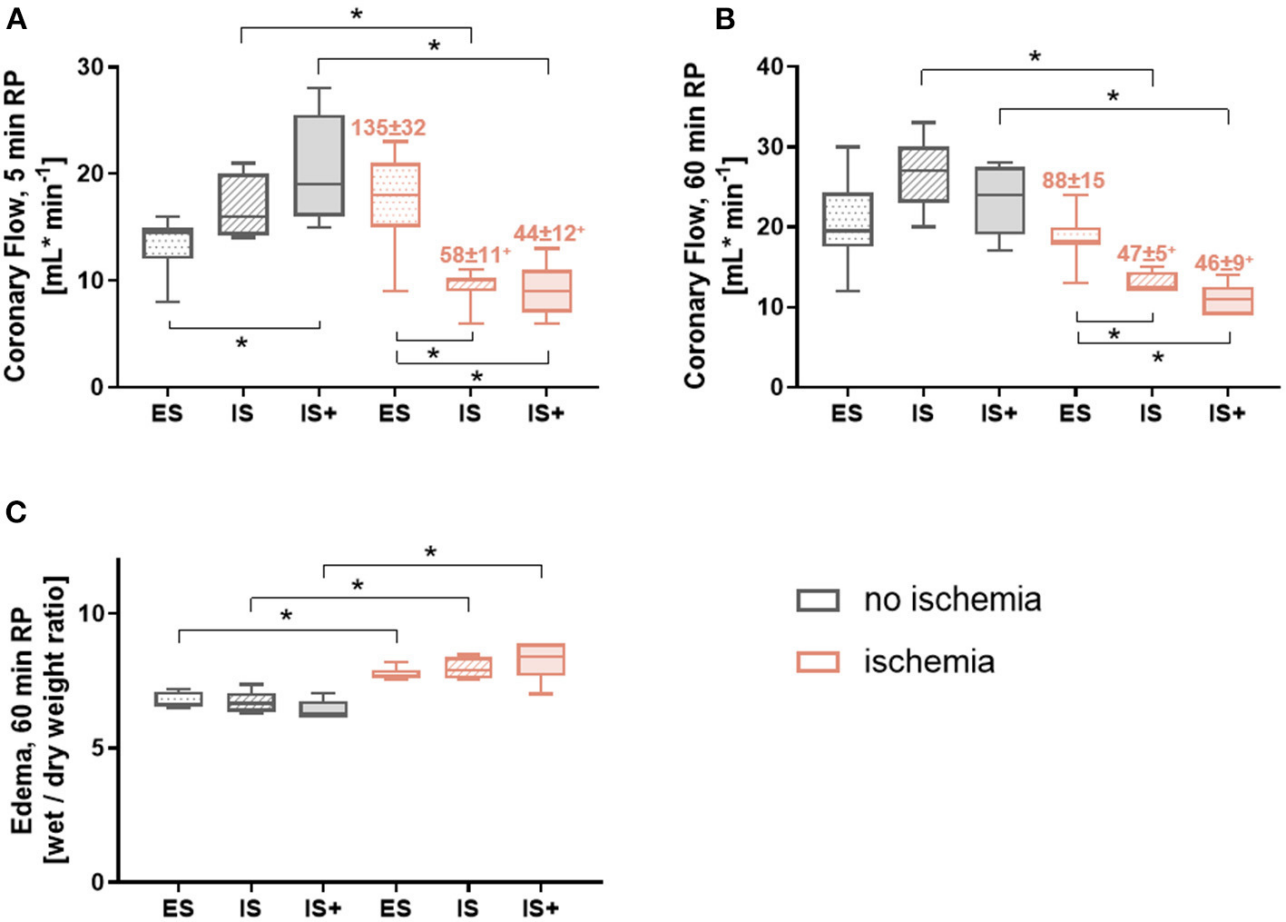
Vascular parameters. **(A)** Coronary flow at 5 min reperfusion **(B)** Coronary flow at 60 min reperfusion **(C)** Tissue water content at 60 min reperfusion. Data are expressed as median, 25–75 percentiles, and range; numbers indicate percentage of corresponding non-ischemic value. *p < 0.05, +*p* < 0.05 vs. ES. ES, *ex-situ* model; IS, *in-situ* model; IS+, *in-situ* model with cardioplegia; RP, reperfusion; *n* = 5–8 per group.

During early reperfusion (5 min), coronary flow was significantly lower in ischemic vs. corresponding non-ischemic hearts in the *in-situ* model (*p* < 0.05 for IS and IS+), but not in the *ex-situ* model (Figure 5A). Among non-ischemic groups, coronary flow was significantly increased in the IS+ hearts compared to the ES hearts (*p* < 0.05; Figure 5A), suggesting greater vasodilation. While in ischemic hearts, coronary flow was significantly decreased in IS and IS+ hearts compared with ES hearts (*p* < 0.05 for both; Figure 5A), and cardioplegic storage did not affect coronary flow.

Similarly, after 60 min reperfusion, coronary flow was significantly lower in ischemic vs. the corresponding non-ischemic hearts in the *in-situ* model (*p* < 0.05 for IS and IS+), but not in the *ex-situ* model (Figure 5B). Among ischemic hearts, coronary flow was significantly lower in the *in-situ* model compared with the *ex-situ* model (*p* < 0.05 for both; Figure 5A), and cardioplegic storage did not affect coronary flow.

Organ edema was significantly increased with ischemia in all the groups compared to their corresponding non-ischemic groups (*p* < 0.05 for all; Figure 5C). No differences among either ischemic groups or non-ischemic groups were observed.

### Circulating Factors

**Figure 6:**
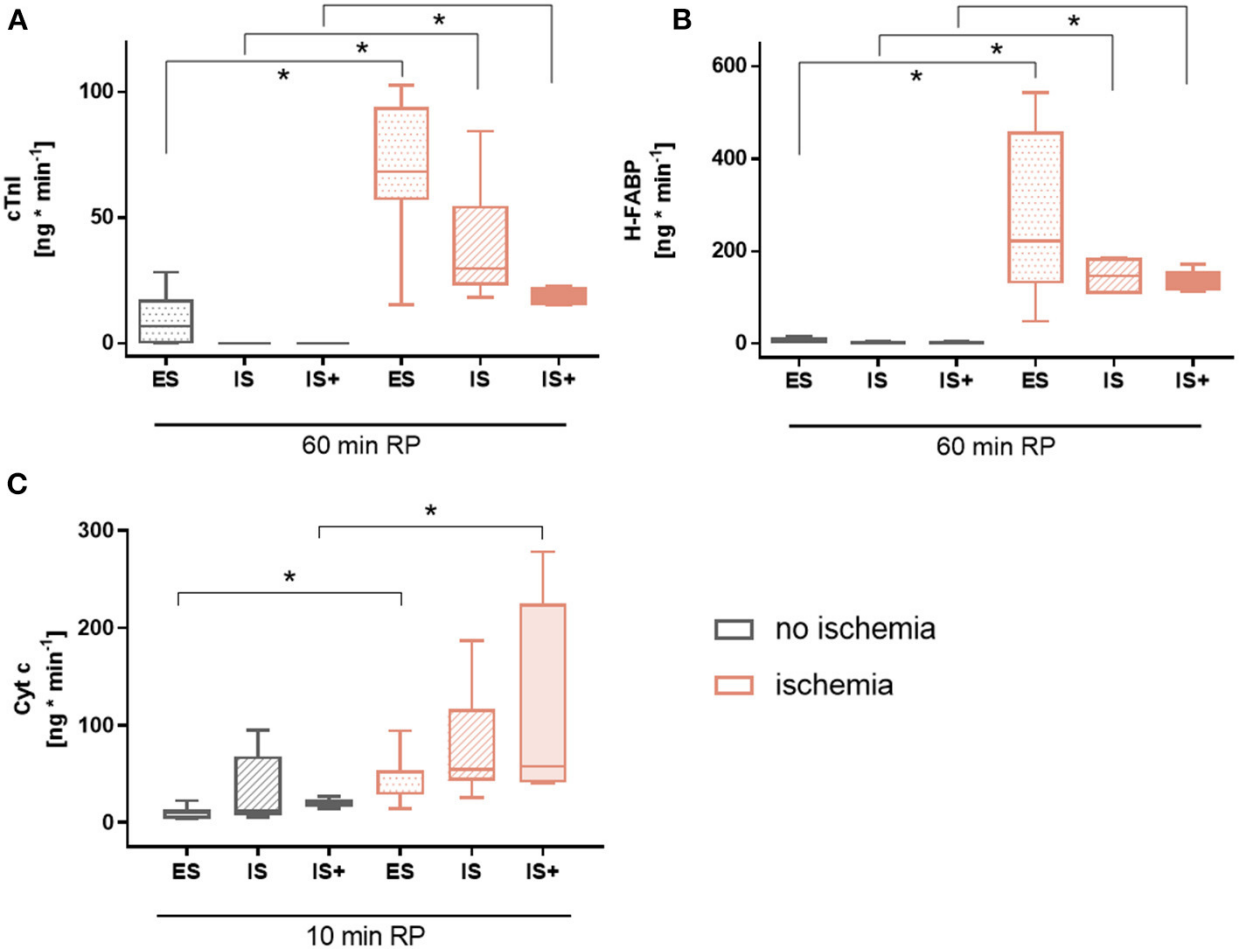
Circulating factors. **(A)** Cardiac troponin I (cTnI) release at 60 min reperfusion **(B)** Heart-type fatty acid binding protein (H-FABP) release at 60 min reperfusion **(C)** Cytochrome c (Cyt c) release at 10 min reperfusion. Data are expressed as median, 25–75 percentiles and range. *p < 0.05. ES, *ex-situ* model; IS, *in-situ* model; IS+, *in-situ* model with cardioplegia; RP, reperfusion; *n* = 5–8 per group.

At 60 min reperfusion, cardiac troponin I was significantly higher in all ischemic groups compared to their corresponding non-ischemic groups (*p* < 0.05 for all), and no differences were observed among ischemic or non-ischemic groups (Figure 6A).

A similar pattern was observed for heart-type fatty acid binding protein (*p* < 0.05 for all; Figure 6B).

Cytochrome c, a marker of mitochondrial damage, tended to be increased in all ischemic groups compared to their corresponding non-ischemic groups, but reached statistical significance only for ES and IS+ hearts (*p* < 0.05 for both; Figure 6C). No differences were observed among ischemic or non-ischemic groups.

## Discussion

For the safe expansion of DCD heart transplantation, preclinical research models are required to help optimize current clinical protocols. Although large animal models, such as porcine models, are necessary to test clinical relevance, studies in smaller animal models, such as rats, are more easily manageable with respect to both technical and financial aspects, thereby allowing only the most promising approaches to proceed with testing in larger animal models. We describe an *in-situ* ischemia rat model of DCD, with characterization of the withdrawal phase, investigation of the effects of a brief period of cold cardioplegia, and comparison of cardiac recovery with a completely *ex-situ* ischemia model. Simulation of withdrawal of life sustaining therapy in the *in-situ* model of DCD induced a hyperdynamic cardiac phase and an elevation in plasma catecholamines before circulatory arrest, which is in agreement with other pre-clinical models of DCD (9, 10, 17). Interestingly, we report that circulating concentrations of FFA are also increased, which is of particular importance as high concentrations of pre-ischemic fatty acids are associated with substantially reduced contractile recovery and greater mitochondrial damage (22). Significantly lower post-ischemic contractile recovery was observed with *in-situ* ischemia (IS) compared to *ex-situ* ischemia (ES). However, the addition of a brief period of cold ischemia with cardioplegia did not further reduce recovery, rather it provided an improvement in left ventricular work compared to *in-situ* ischemia without cardioplegia. However, cardioplegia was not able to rescue decreased post-ischemic coronary flow, which was significantly lower in both *in-situ* conditions vs. ES, suggesting greater endothelial dysfunction/damage. Importantly, no difference between models was observed for markers of cell death or mitochondrial damage. In summary, we conclude that the choice of experimental model can affect cardiac recovery in a variable-specific manner and that models for cardiac DCD require careful selection according to the particular research focus (Table 3).

**Table 3:** Advantages and disadvantages of an *ex-situ* and *in-situ* ischemia model of DCD.

	**Advantages**	**Disadvantages**	**Suggested application**
*Ex-situ* ischemia model	Better control of experimental conditions (e.g., pre-ischemic conditions; clearly defined duration of warm ischemia)	Relevance of findings need to be evaluated in the context of scientific question and clinical DCD conditions	Mechanistic studies investigating pathophysiology of cardiac IRI or molecular effects of therapeutic interventions and/or physical or (bio)chemical conditions
*In-situ* ischemia model	Increased clinical relevance in the context of DCD	Risk of increased variability due to less tightly controlled conditions; no consensus for start or end of FWIT in clinical DCD protocols	Studies evaluating strategies to improve aspects of DCD clinical protocols such cardioprotection, storage conditions or graft evaluation

*DCD, donation after circulatory death; FWIT, functional warm ischemic time; IRI, ischemia-reperfusion injury*.

In the *in-situ* model of DCD, hearts are exposed to variable conditions prior to procurement, which can impact on cardiac recovery. Upon simulation of WLST, hearts developed a hyperdynamic phase characterized by increased heart rate and pulse pressure, whereas blood oxygen saturation and mean peak systolic pressure continuously decreased. This hyperdynamic phase was previously described in other preclinical models of DCD (9, 10, 17). As the donor heart initially works under rapidly developing hypotension and hypoxia, instantaneous systemic responses to restore blood pressure are stimulated, including catecholamine release. Accordingly, we detected increased concentrations of catecholamines and circulating FFA in plasma of rats at the end of the withdrawal, as well as the ischemic phase. Besides influencing cardiac rhythm, contractility and increasing intracranial pressure (28), catecholamines have been shown in experimental settings to cause lactate accumulation, ATP depletion, myocyte damage, and contraction band necrosis (29, 30). Furthermore, catecholamines also trigger adaptations in cardiac metabolism. Despite the tendency to decrease between withdrawal of treatment and heart procurement, concentrations of FFA remained high compared with physiologic values (0.4 mM) (31). Sympathetic stimulation induces lipolysis of stored glycerides in adipocytes and increases concentrations of circulating FFA. This is of particular importance as FFA have been demonstrated to exacerbate reperfusion injury (22). Upon reperfusion, as soon as oxygen is reintroduced to the tissue, oxidation of FFA is stimulated and increases mitochondrial ratios of NADH/NAD^+^ and acetyl-CoA/CoA, both of which activate pyruvate dehydrogenase kinase. Activated pyruvate dehydrogenase kinase inhibits pyruvate dehydrogenase, the rate limiting enzyme of glucose oxidation. Reduced glucose oxidation at the beginning of reperfusion results in uncoupling between glycolysis and glucose oxidation and to a greater conversion of pyruvate to lactate. Acidification of the cytoplasm can further aggravate calcium overload and reperfusion injury (32). Another cause of high concentrations of circulating FFA is heparin. In DCD, heparin concentrations used are several times higher compared with ordinary clinical anticoagulation (>300 units/kg vs. ~80 units/kg) (33). Furthermore, in agreement with other studies (34), heparinization was required in this model to achieve effective heart reperfusion and prevent from clot formation. In addition, heparin activates lipoprotein lipase at the endothelial surface and stimulates the release of FFA from circulating lipoproteins (31). Our results do not permit the distinction between effects of heparinization and catecholamine release on circulating FFA. However, as the circulating FFA were already high during pre-withdrawal of life sustaining therapy, when measured catecholamine concentrations were very low, it is likely that initial free fatty acid release was induced by heparin and that subsequent catecholamine-mediated lipolysis was masked. Additionally, the increasing pattern of catecholamines in parallel with the decreasing pattern of FFA during the withdrawal phase also suggest that FFA release was likely initially induced by heparin addition. It is technically difficult to determine circulating FFA accurately in heparinized blood as lipolysis may continue after sample collection, and an overestimation in our samples of 20–50% can be expected (35). However, taking this into consideration, the FFA levels measured during the withdrawal period are still substantially higher than physiologic levels.

As systemic changes during the withdrawal phase and ischemia *in situ* can influence post-ischemic cardiac recovery, it is essential that they are reflected in experimental models. Over the last years, we developed an *ex-situ* model that includes specific DCD-relevant conditions (3–6, 8) with, e.g., tight temperature control during warm ischemia and the use of supraphysiologic perfusate FFA concentrations (1.2 mM palmitate) prior to ischemia. Nonetheless, some differences in post-ischemic recovery between *in-situ* and *ex-situ* models persist and may be explained by systemic changes during withdrawal and ischemia *in situ*. In general, post-ischemic recovery was higher in the *ex-situ* vs. *in-situ* model. In previous work with our *ex-situ* model, functional recovery deteriorated only significantly after 27 min ischemia, with a steep decline between 27 and 33 min ischemia (5, 6). In the *in-situ* model, before 27 min FWIT (starting with peak systolic pressure <50 mmHg), the entire body and heart are exposed to hypoxia during the withdrawal period as demonstrated by low oxygen saturation, supraphysiologic concentrations of circulating lactate and CO_2_ at FWIT. Pre-ischemic hypoxia may be detrimental through the reduction of ATP production via oxidative phosphorylation, in parallel with increased lactate production and associated acidosis. It is known that hypoxia may also exert protective effects, as it could stimulate pro-survival pathways activated by pre-conditioning; however, these protocols generally require intermittent application by several cycles of ischemia and reperfusion (36). Therefore, the additional exposure to hypoxia/ischemia could be a reason for the lower post-ischemic recovery in IS vs. ES hearts. Further damage may also result from increased levels of catecholamines, as catecholamine storms have been associated with detrimental organ function in brain dead donors (37, 38).

Although the cold, cardioplegic storage used in this study adds a potentially damaging period of cold ischemia, our findings indicate that it may provide some cardioprotection, potentially via the cold, coronary flush and addition of protective agents. Although not significantly different, tendencies for improved contractile function and lower release of the cell death marker cardiac troponin I were visible in IS+ vs. IS groups. EPO, a component of the cardioplegia, has been shown to be cardioprotective, through the activation of the pro-survival Akt signaling pathway and decreased cell death (39–41). GTN is a nitric oxide donor and a widely used drug to treat ischemic heart disease (42). The addition of GTN and EPO in Celsior cardioplegia was cardioprotective in rat and pig heart models (43, 44). EPO and GTN may therefore contribute to the trend of improved recovery in IS+ vs. IS hearts. Although we see improved contractile function and reduced release of cell death markers in IS+ vs. IS groups, cardioprotection could potentially be further optimized with use of a preservation solution that is better suited for DCD organs than St. Thomas' N°2, for example with a normokalemic and hypocalcemic solution.

In this study, ischemia is associated with substantial changes in coronary flow. In the absence of ischemia, coronary flow at 5 min reperfusion was higher in IS+ vs. ES hearts. This higher coronary flow may result from the hyperemic response since IS+ hearts were subjected to a short period of ischemia during procurement and cannulation, and cold storage when used, immediately prior to reperfusion. During ischemia, the heart produces vasoactive compounds, such as adenosine, leading to the vasodilation of the arterioles, the reduction of vascular resistance and elevated coronary flow upon reperfusion, resulting in hyperemia (45). Furthermore, with the cardioplegia, IS+ hearts received both GTN, a potent vasodilator via donation of nitric oxide (46), and EPO, which has been shown to upregulate endothelial nitric oxide synthase -derived nitric oxide production by increasing phosphorylation and expression through PI3-kinase/Akt (47). Thus, hyperemia, as well as GTN and EPO supplementation, likely contribute to increased coronary flow in IS+ vs. ES hearts in the absence of warm ischemia. Post-ischemic coronary flow was significantly lower in IS and IS+ vs. ES hearts at both early and late reperfusion time points. Although the mechanism for this is not entirely clear, it is unlikely to result from differences in edema-induced flow reductions since all post-ischemic groups demonstrated similar levels of edema. Contractile function tended to be greater in ES hearts, potentially leading to a greater metabolic demand, which could stimulate coronary flow. Alternatively, one might speculate that reductions in coronary flow in IS and IS+ hearts may be a result of greater exposure to adhesion molecules promoting platelet aggregates, platelet-leukocyte aggregates and erythrocyte aggregates in hearts undergoing ischemia *in situ*, which could block the capillaries and lead to no-reflow (48).

Although the *in-situ* withdrawal period and ischemia represent major differences from the *ex-situ* model, additional differences are present between models that could influence outcomes. For example, our *in-situ* model requires ante-mortem heparinization, whereas no heparin was used in the *ex-situ* model. Pre-ischemic heparin has been reported to reduce ischemia-reperfusion injury in rabbit hearts (49, 50); however, we generally observed reduced recovery in hearts exposed to heparin. Furthermore, isolated heart preparations are recognized to deteriorate with time (51) and our *in-situ* ischemia hearts were maintained in the isolated system for approximately half the time of those from the *ex-situ* model, which would be expected to result in lower post-ischemic function in ES vs. IS and IS+ hearts, which is opposite to our observations. Lastly, due to the *ante-mortem* surgical intervention (cannulation of carotid artery), the anesthetic protocol for the *in-situ* model (ketamine, xylazine, acepromazine) differs from that of the *ex-situ* model (ketamine, xylazine).

### Limitations

This study has several limitations. The models we used do not account for neurologic injury, which is almost always present in DCD donors, and could potentially lead to remote preconditioning. In our *in-situ* model, WLST is simulated by dissection of the animal's diaphragm (asphyxiation model of DCD), which may result in a shorter duration of the period between WLST and circulatory arrest compared with human DCD donors who may display remnants of spontaneous respiration (52). Additionally, due to the fact that rats did not undergo intermittent positive pressure ventilation during general anesthesia, hypercapnia and respiratory acidosis were present before WLST, which can be avoided in human DCD donors. The 15-min cold static period implemented in the group of hearts treated with St. Thomas' N°2 cardioplegic solution is a relatively short period in the clinical setting for preparation of the heart and/or the *ex-situ* perfusion machine. Future work could include additional studies to better characterize the loss of myocardial function in DCD by measuring, for example, infarct size; as well as studies with increased sample size to confirm the relevance of our results.

## Conclusions

In this study, we characterized an *in-situ* rat model of DCD. DCD grafts were subjected to hypoxia, hemodynamic changes, acidosis, and increased concentrations of catecholamines and circulating FFA before and during ischemia, which highlights the suitability of this model for further research. *In-situ* conditions were more detrimental for hearts vs. *ex-situ* conditions in terms of post-ischemic contractility and relaxation rates, and cardiac output. The application of cardioplegia tended to abrogate differences compared to ES for cardiac function, with the exception of cardiac output and coronary flow. However, given the complexity of WLST and ischemia *in situ*, future studies toward the optimization of clinical DCD protocols will certainly benefit from preclinical models, which incorporates the effects of systemic, and blood-borne changes upon WLST.

## Data Availability Statement

The raw data supporting the conclusions of this article will be made available by the authors, without undue reservation.

## Ethics Statement

The animal study was reviewed and approved by Ethics Committee for Animal Experimentation, Berne, Switzerland (Veterinärdienst des Kantons Bern).

## Author Contributions

MA, NM-C, and RW contributed to all aspects of this manuscript including study planning and design, model development, performing experiments, data collection and analysis, and preparation of the manuscript. AJ participated in study planning and design, model development, performing experiments, and data collection and analysis. DC and SL participated in study planning and design, model development, data analysis, and preparation of the manuscript. TC participated in data analysis and preparation of the manuscript. All authors contributed to the article and approved the submitted version.

## Conflict of Interest

The authors declare that the research was conducted in the absence of any commercial or financial relationships that could be construed as a potential conflict of interest.

## Abbreviations

DCD: donation after circulatory death
dP/dt max: maximum first derivative of left ventricular pressure
dP/dt min: minimum first derivative of left ventricular pressure
EPO: erythropoietin
ES: ischemic *ex-situ* model
FFA: free fatty acids
FWIT: functional warm ischemic time
GTN: glyceryl trinitrate
IS: ischemic *in-situ* model
IS+: ischemic *in-situ* model with cardioplegia
min: minutes
PCO_2_: partial carbon dioxide pressure
PO_2_: partial oxygen pressure
WLST: withdrawal of life sustaining therapy.
