# Supplementary material for: Comparison of Experimental Rat Models in Donation After Circulatory Death (DCD): in-situ vs. ex-situ Ischemia
Source: Front Cardiovasc Med. 2021 Jan 13;7:596883. doi: 10.3389/fcvm.2020.596883 (PMC7838125; doi:10.3389/fcvm.2020.596883)
Supplement: Supplementary file 1 [file Table_1.DOCX]

Supplementary Tables

|  |  | *NI-IS* | *NI-IS+* | *I-IS* | *I-IS+* | *All hearts* |
| --- | --- | --- | --- | --- | --- | --- |
|  | **WLST – FWIT (min)** | 1.1 ± 0.3 | 0.6 ± 0.4 | 1.3 ± 1.0 | 1.1 ± 0.3 | 1.0 ± 0.6 |
|  | **FWIT – CA (min)** |  |  | 1.7 ± 1.0 | 2.1 ± 0.8 | 1.9 ± 0.9 |
|  | **WLST – CA (min)** |  |  | 3.0 ± 1.4 | 3.2 ± 0.8 | 3.1 ± 1.1 |
|  |  |  |  |  |  |  |
| pre-W | **PSP (mmHg)** | 95 ± 16 | 93 ± 10 | 107 ± 10 | 104 ± 5 | 100 ± 12 |
|  | **HR (bpm)** | 224 ± 9 | 234 ± 43 | 249 ± 37 | 255 ± 12 | 241 ± 30 |
|  | **PP (mmHg)** | 29 ± 16 | 17 ± 11 | 31 ± 13 | 29 ± 5 | 26 ± 12 |
|  | **PO_2_ (mmHg)** | 246 ± 63 | 175 ± 10 | 233 ± 91 | 181 ± 59 | 212 ± 72 |
|  | **PCO_2_ (mmHg)** | 78 ± 27 | 61 ± 5 | 58 ± 11 | 57 ± 8 | 63 ± 16 |
|  | **pH** | 7.3 ± 0.1 | 7.3 ± 0.0 | 7.3 ± 0.0 | 7.3 ± 0.0 | 7.2 ± 0.1 |
|  | **lactate (mM)** | 0.9 ± 0.6 | 0.8 ± 0.5 | 0.5 ± 0.1 | 1.0 ± 0.4 | 0.8 ± 0.4 |
|  | **calcium (mM)** | 1.4 ± 0.1 | 1.6 ± 0.1 | 1.3 ± 0.1 | 1.6 ± 0.1 | 1.5 ± 0.2 |
|  | **potassium (mM)** | 5.6 ± 1.3 | 5.8 ± 1.0 | 4.9 ± 0.6 | 4.7 ± 0.2 | 5.2 ± 0.9 |
|  | **sodium (mM)** | 153 ± 24 | 159 ± 20 | 141 ± 5 | 138 ± 3 | 147 ± 16 |
|  | **chloride (mM)** | 106 ± 3 | 105 ± 2 | 106 ± 4 | 106 ± 4 | 106 ± 3 |
|  | **glucose (mM)** | 17 ± 6 | 19 ± 1 | 17 ± 2 | 19 ± 1 | 18 ± 3 |
|  | **adrenaline (ng * mL^-1^)** | 0.1 ± 0.1 | 0.2 ± 0.1 | 0.1 ± 0.1 | 3.7 ± 8.3 | 1.0 ± 4.0 |
|  | **noradrenaline (ng * mL^-1^)** | 0 | 0 | 0 | 0 | 0 |
|  | **free fatty acids (mM)** | 2.3 ± 0.6 | 2.1 ± 0.5 | 2.6 ± 0.7 | 2.1 ± 0.6 | 2.3 ± 0.6 |
|  |  |  |  |  |  |  |
| HD | **HR (bpm)** | 284 ± 41 | 284 ± 36 | 311 ± 37 | 303 ± 27 | 295 ± 35 |
|  | **PP (mmHg)** | 37 ± 20 | 31 ± 19 | 37 ± 15 | 43 ± 9 | 37 ± 16 |
|  |  |  |  |  |  |  |
| FWIT start | **PSP (mmHg)** | 50 ± 0 | 50 ± 0 | 50 ± 0 | 49 ± 1 | 50 ± 1 |
|  | **HR (bpm)** | 184 ± 57 | 196 ± 98 | 239 ± 59 | 254 ± 70 | 218 ± 73 |
|  | **PP (mmHg)** | 22 ± 10 | 16 ± 9 | 22 ± 8 | 24 ± 3 | 21 ± 8 |
|  | **PO_2_ (mmHg)** | 17 ± 8 | 13 |  |  | 16 ± 6 |
|  | **PCO_2_ (mmHg)** | 92 ± 15 | 84 ± 8 |  |  | 94 ± 23 |
|  | **pH** | 7.1 ± 0.1 | 7.2 ± 0.0 |  |  | 7.2 ± 0.1 |
|  | **lactate (mM)** | 1.0 ± 0.5 | 1.1 ± 0.5 |  |  | 1.1 ± 0.5 |
|  | **calcium (mM)** | 1.5 ± 0.1 | 1.7 ± 0.3 |  |  | 1.7 ± 0.4 |
|  | **potassium (mM)** | 6.5 ± 0.7 | 7.2 ± 1.0 |  |  | 6.9 ± 0.9 |
|  | **sodium (mM)** | 141 ± 4 | 148 ± 8 |  |  | 145 ± 7 |
|  | **chloride (mM)** | 104 ± 3 | 102 ± 1 |  |  | 103 ± 2 |
|  | **glucose (mM)** | 14 ± 8 | 18 ± 0 |  |  | 16 ± 5 |
|  | **adrenaline (ng * mL^-1^)** | 0.2 ± 0.1 | 0.1 ± 0.2 |  |  | 0.2 ± 0.2 |
|  | **noradrenaline (ng * mL^-1^)** | 0.2 ± 0.2 | 0.3 ± 0.3 |  |  | 0.2 ± 0.3 |
|  | **free fatty acids (mM)** | 1.7 ± 0.8 | 0.9 ± 0.3 |  |  | 1.2 ± 0.7 |
|  |  |  |  |  |  |  |
| Circulatory arrest | **PSP (mmHg)** |  |  | 23 ± 8 | 16 ± 4 | 20 ± 7 |
|  | **HR (bpm)** |  |  | 166 ± 72 | 187 ± 64 | 176 ± 66 |
|  | **PP (mmHg)** |  |  | 3 ± 0 | 3 ± 0 | 3 ± 0 |
|  | **PO_2_ (mmHg)** |  |  | 15 ± 4 | 22 ± 10 | 19 ± 8 |
|  | **PCO_2_ (mmHg)** |  |  | 97 ± 6 | 90 ± 15 | 93 ± 12 |
|  | **pH** |  |  | 7.1 ± 0.0 | 7.1 ± 0.1 | 7.1 ± 0.0 |
|  | **lactate (mM)** |  |  | 3.9 ± 1.0 | 3.7 ± 1.3 | 3.8 ± 1.1 |
|  | **calcium (mM)** |  |  | 1.5 ± 0.0 | 1.6 ± 0.1 | 1.6 ± 0.1 |
|  | **potassium (mM)** |  |  | 7.1 ± 0.5 | 6.8 ± 0.7 | 6.9 ± 0.6 |
|  | **sodium (mM)** |  |  | 139 ± 5 | 138 ± 4 | 139 ± 4 |
|  | **chloride (mM)** |  |  | 103 ± 2 | 104 ± 2 | 104 ± 2 |
|  | **glucose (mM)** |  |  | 21 ± 2 | 19 ± 1 | 20 ± 2 |
|  | **adrenaline (ng * mL^-1^)** |  |  | 7.8 ± 9.3 | 9.2 ± 9.3 | 8.8 ± 8.6 |
|  | **noradrenaline (ng * mL^-1^)** |  |  | 1.4 ± 1.0 | 1.7 ± 1.4 | 1.5 ± 1.1 |
|  | **free fatty acids (mM)** |  |  | 2.2 ± 1.0 | 0.8 ± 0.1 | 1.5 ± 1.0 |

**Table S1. Measurements during *in-situ* / withdrawal phase.** CA, circulatory arrest; FWIT, functional warm ischemia start; HD, hyperdynamic phase; HR, heart rate; I-IS, ischemic *in-situ* model; I-IS+, ischemic *in-situ* model with cardioplegia; NI-IS, non-ischemic *in-situ* model; NI-IS+, non-ischemic *in-situ* model with cardioplegia; PCO_2_, partial carbon dioxide pressure; PO_2_, partial oxygen pressure; PP, pulse pressure; pre-W, pre-withdrawal of life sustaining therapy; PSP, peak systolic pressure; WLST, withdrawal of life sustaining therapy.
