# Supplementary figures and images for: Comparison of Experimental Rat Models in Donation After Circulatory Death (DCD): in-situ vs. ex-situ Ischemia
Source: Front Cardiovasc Med. 2021 Jan 13;7:596883. doi: 10.3389/fcvm.2020.596883 (PMC7838125; doi:10.3389/fcvm.2020.596883)

*Supplementary Figures*

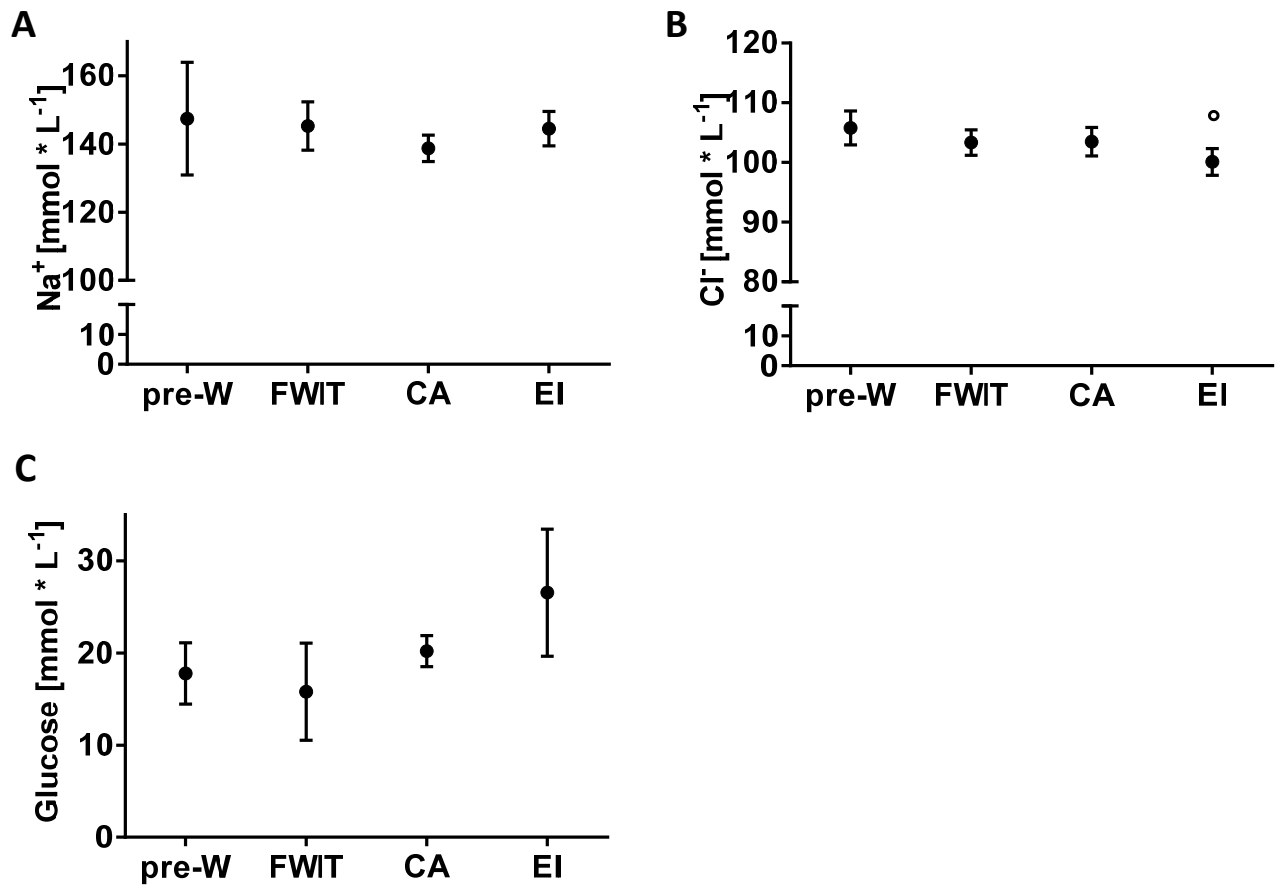

Figure S1.

Supplement: Supplementary file 2 [file Data_Sheet_1.PDF]

# Supplementary Figures

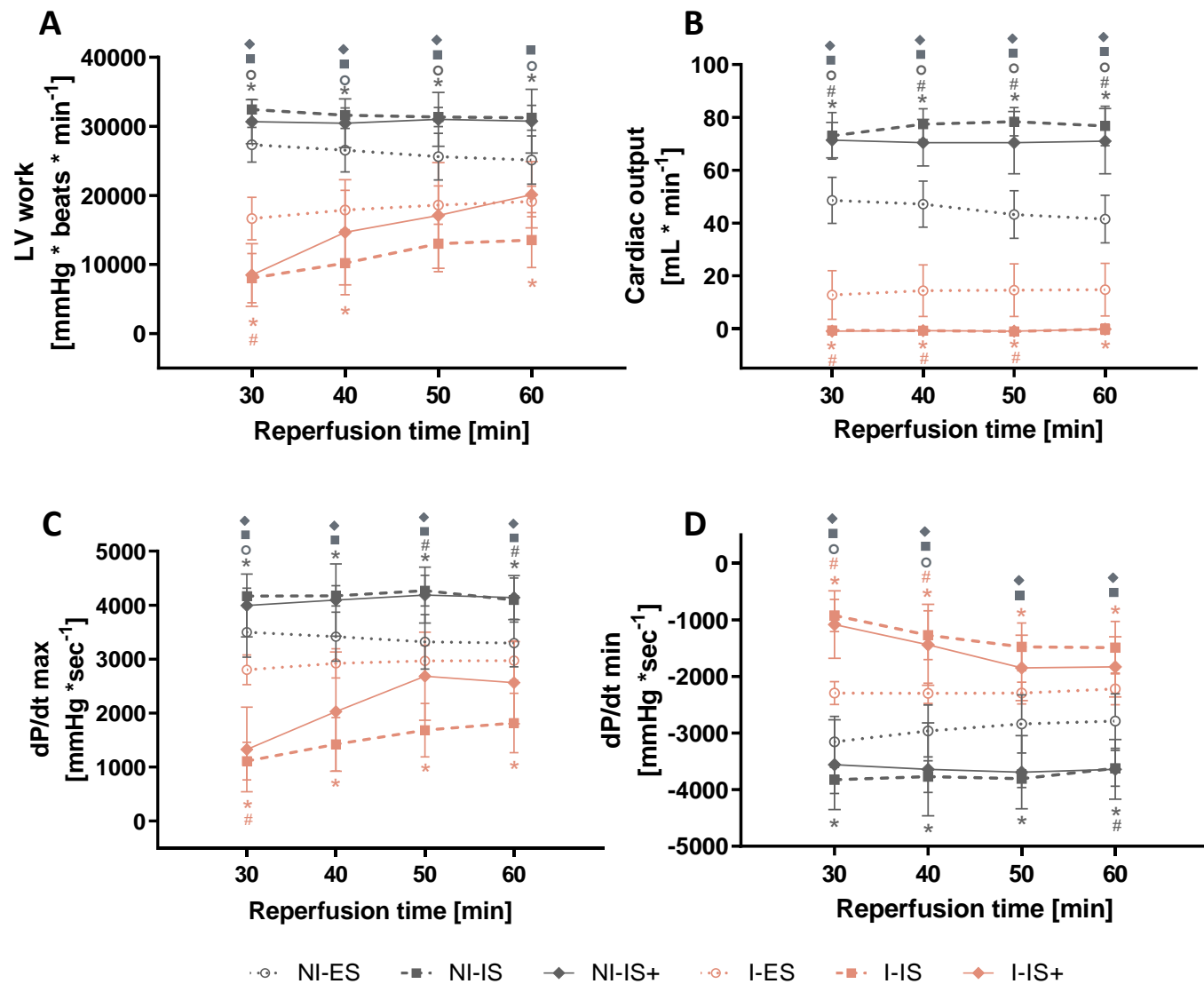

Figure S2.

Supplement: Supplementary file 3 [file Data_Sheet_2.PDF]
