# Supplementary material for: Comparison of Experimental Rat Models in Donation After Circulatory Death (DCD): in-situ vs. ex-situ Ischemia
Source: Front Cardiovasc Med. 2021 Jan 13;7:596883. doi: 10.3389/fcvm.2020.596883 (PMC7838125; doi:10.3389/fcvm.2020.596883)
Supplement: Supplementary file 4 [file Data_Sheet_3.PDF]

Supplementary Figures

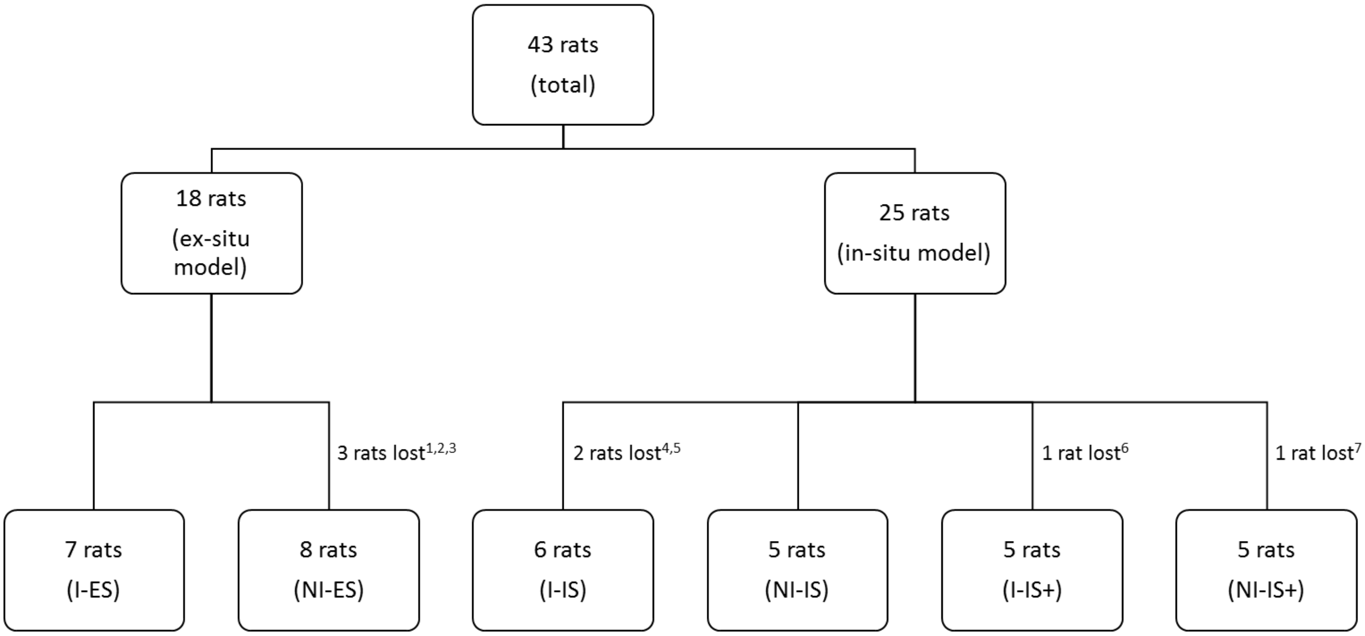

1. Catheter not working: no functional data; 2. Flow meter not working: no flow data, 3. Fibrillation & arrhythmia in reperfusion, 4. Unsuccessful aorta cannulation, 5. Fibrillation & arrhythmia in reperfusion, 6. Ischemic temperature too high, 7. unsuccessful cardioplegia delivery

Figure S3.
