# Supplementary material for: Comparison of Experimental Rat Models in Donation After Circulatory Death (DCD): in-situ vs. ex-situ Ischemia
Source: Front Cardiovasc Med. 2021 Jan 13;7:596883. doi: 10.3389/fcvm.2020.596883 (PMC7838125; doi:10.3389/fcvm.2020.596883)
Supplement: Supplementary file 5 [file Data_Sheet_4.docx]

Supplementary Methods

# Measurement of blood gas and circulating factors

## Measurements in both blood and perfusate samples

Partial O_2_ and CO_2_ pressures, electrolytes, pH and lactate were measured using a Cobas b 123 blood gas analyzer (Roche, Basel, Switzerland). Lactate concentrations below the detection limit (<1 mM) were additionally measured with a lactate assay (Sigma-Aldrich, Missouri, USA).

## Circulating factors in plasma samples

Concentrations of catecholamines were measured using the Epinephrine/Norepinephrine ELISA kit (Abnova, Taipeh City, Taiwan). Circulating free fatty acids were measured using the Free Fatty Acid Quantitation Kit (Sigma–Aldrich, Missouri, USA).

## Circulating factors in perfusate samples

Mitochondrial cytochrome c (Cyt c) release was measured using the Quantikine ELISA kit for Rat/Mouse Cyt-c (R&D Systems; Minneapolis, USA). Myocardial heart-type fatty acid binding protein (H-FABP) release was measured using the rat H-FABP ELISA kit (Life Diagnostics, Pennsylvania, USA). Myocardial cardiac troponin I (cTnI) release was measured using the rat cardiac Troponin-I ELISA kit (Life Diagnostics, Pennsylvania, USA). Release of factors were calculated as:

$$Cyt c=CF(t_{x})\times{(C}_{CE}(t_{x})-C_{P}(t_{x})) H-FABP/ cTnI= \frac{C_{P}(t_{x})\times V_{P}(t_{x})}{t_{x}}$$

C: concentration; CE: coronary effluent; CF: coronary flow; P: circulating perfusate; t_x_: time point of interest; V: total perfusate volume

# Measurement of tissue water content

Tissue water content was measured by calculating the wet/dry weight ratio. Powdered tissue samples were weighed before and after drying in an oven at 60 °C for 24 hours.

Supplementary Figure Legends

**Figure S1. Blood parameters during *in-situ* /withdrawal phase.** A) blood sodium (Na+) B) blood chloride (Cl-) C) blood glucose. CA, circulatory arrest; EI, end-ischemia; FWIT, functional warm ischemia start; pre-W, pre-withdrawal of life sustaining therapy. °*p*<0.05 vs. corresponding previous time point; n = 8-21 / time point.

**Figure S2.** **Post-ischemic cardiac function.** A) LV work (left ventricular work: heart rate*developed pressure) B) CO (cardiac output) C) dP/dt max (maximum first derivative of LV pressure) D) dP/dt min (minimum first derivative of LV pressure). * *p*<0.05, NI-ES vs NI-IS; # *p*<0.05, NI-ES vs NI-IS+; * *p*<0.05, I-ES vs I-IS; # *p*<0.05, I-ES vs I-IS+; ° *p*<0.05, NI-ES vs I-ES; ◼ *p*<0.05, NI-IS vs I-IS; ◆ *p*<0.05, NI-IS+ vs I-IS+. I-ES, ischemic *ex-situ* model; I-IS, ischemic *in-situ* model; I-IS+, ischemic *in-situ* model with cardioplegia; NI-ES, non-ischemic *ex-situ* model; NI-IS, non-ischemic *in-situ* model; NI-IS+, non-ischemic *in-situ* model with cardioplegia; Data are expressed as mean ± SD; n = 5–8 per group.

**Figure S3.** Flow chart depicting number of rats in each experimental group.
